# Mechanical Nociceptive Threshold, Tissue Alterations and Horn Growth in Calves after Injection of Isoeugenol or Clove Oil under the Horn Bud

**DOI:** 10.3390/ani11030828

**Published:** 2021-03-15

**Authors:** Anna Juffinger, Julia Schoiswohl, Anna Stanitznig, Reinhild Krametter-Frötscher, Thomas Wittek, Susanne Waiblinger

**Affiliations:** 1 Institute of Animal Welfare Science, Department for Farm Animals and Veterinary Public Health, University of Veterinary Medicine, Veterinärplatz 1, 1210 Vienna, Austria; Anna.Juffinger@vetmeduni.ac.at; 2 University Clinic for Ruminants, Department for Farm Animals and Veterinary Public Health, University of Veterinary Medicine, Veterinärplatz 1, 1210 Vienna, Austria; Julia.Schoiswohl@vetmeduni.ac.at (J.S.); Anna.Stanitznig@vetmeduni.ac.at (A.S.); Reinhild.Krametter@vetmeduni.ac.at (R.K.-F.); Thomas.Wittek@vetmeduni.ac.at (T.W.)

**Keywords:** disbudding, cattle, pain, nociception, horn growth, welfare

## Abstract

**Simple Summary:**

Hot-iron disbudding of calves is a common but painful practice. We investigated the injection of clove oil or isoeugenol under the horn bud as a potential alternative. Compared to hot-iron disbudding (with local anesthesia), pain sensitivity increased much less and for a shorter time in isoeugenol-injected calves. After injection of clove oil, the differences to hot-iron disbudding were smaller, and there was a high occurrence of swellings of the eyelids. Neither injection was as effective as the use of a hot iron in preventing horn growth. In sum, the injection of clove oil is not recommendable, and regarding the use of isoeugenol further research is needed, in particular on possibilities to improve its effectiveness.

**Abstract:**

Disbudding of calves is a common, painful intervention. Due to cytotoxic and anesthetic properties, the injection of clove oil or its component isoeugenol may be less detrimental to animal welfare. We investigated mechanical nociceptive threshold (MNT), possible tissue alterations and horn growth for up to 12 weeks after injection of 1.5 mL clove oil (CLOV), isoeugenol (ISO) or saline (CON) or after hot-iron disbudding (BURN; with local anesthesia and sedation, *n* = 10/treatment). MNT was measured using von Frey filaments and a pressure algometer at four locations around the horn bud. There was a treatment*time point interaction (linear mixed model, *p* < 0.05). MNT decreased most strongly and for the longest time for BURN in most calves at least for 3 weeks. For ISO, the decrease was less distinct and most calves’ values returned to baseline after 1–2 weeks. MNT in CLOV was intermediate, with decreased values up to 3 weeks in some animals. 12 weeks after the treatment, horn growth was prevented in about 50% of the horns in CLOV and ISO. Tissue alterations such as swellings of the eyelids often occurred in CLOV, but less so in ISO. Our results suggest that injection of isoeugenol causes less pain and thus seems to be beneficial compared to hot-iron disbudding, while clove oil was not advantageous. Regarding the effectiveness of isoeugenol to prevent horn growth, more studies are needed.

## 1. Introduction

The disbudding of calves is a routine intervention in dairy cattle farming. In total, 94.3% in the USA [1] and 81% of the dairy cattle in Europe [2] are disbudded or dehorned. The most frequently used argument for disbudding calves is the protection against injuries to conspecifics and humans [2,3]. Although the risk of injuries in herds of horned dairy cows is controllable and highly management-dependent [2,4], the keeping of horned dairy cows in loose housing is uncommon and most farmers prefer hornless cows.

The most common method in Europe and the USA for disbudding of calves is the use of a hot iron; other methods are using a scoop, caustic paste, rubber rings or surgical removal of the horn buds [1,2,3]. All of these methods cause extensive wounds that need several weeks to heal and induce pain not only during the procedure itself but also in the longer term, impairing the well-being of calves [5,6]. For example, the mechanical nociceptive threshold (MNT), i.e., the amount of pressure an individual tolerates before withdrawing [7,8], is lowered after disbudding, in some calves for up to 15 weeks, indicating hyperalgesia or allodynia [6]. Changes in MNT associated with disbudding can either be assessed by using a pressure algometer (PA) (e.g., [5,7,9,10]) or von Frey filaments (vFF) [6,11]. These two devices differ in their measurement range and thus sensitivity to detect changes in MNT. In most studies, MNT was measured within the first hours after disbudding, assessing acute pain [7,9,10]. Only a few recent studies [5,6] used MNT for the assessment of pain for several weeks or even months after disbudding. These studies showed that the pain due to hot-iron disbudding can persist at least until re-epithelialization which takes on average 9 weeks [5] or even up to 15 weeks [6], which supports the assumption that the calves’ welfare is compromised for a long time and that there is a need for more welfare-friendly methods as long as the practice of disbudding is not abandoned completely.

Clove essential oil (*Caryophylli aetheroleum*) has anesthetic, analgesic, antibacterial and cytotoxic properties mainly associated with its principal active component eugenol [12,13]. Similar properties have also been shown for the eugenol-isomer isoeugenol [14]. Due to the latter properties, the injection of clove oil under the horn bud was investigated as potential alternative to hot-iron disbudding in calves [10,15,16,17] and goats [18,19,20,21]. In two studies with very small sample sizes, clove oil prevented horn growth completely in calves and goats [15,18], whereas in larger studies on calves and goats, complete prevention was only seen in 87% of calves after 6 months [16] and in 13% of goats after 5 months [21]. Calf behavior (e.g., less head shaking and head rubbing) indicated initially less pain after injection of clove oil when compared to hot-iron disbudding, although 48 h after the treatment the MNT was similar in both treatments [10]. However, neither the MNT development over the first 48 h nor its longer-term effects were investigated. In addition, no study so far compared the injection of isoeugenol with hot-iron disbudding. Isoeugenol may be superior to clove oil due to its standardized high concentration, the lack of additional constituents that can induce hypersensitivity [22] and its common usage for anesthesia in fish [23]. Indeed, in a study comparing injection of clove oil and isoeugenol under the horn bud of calves and goat kids, the course of the MNT suggested less pain after use of isoeugenol [24]. However, only the first 24 h were investigated and no control treatments were used.

Thus, the aim of our study was to compare the injection of clove oil or isoeugenol under the horn bud of calves, with the injection of saline and hot-iron disbudding with respect to the MNT until three weeks after the treatment and horn growth, wound healing and tissue alterations up to 12 weeks. We hypothesized that injection of clove oil and isoeugenol lead to a lower decrease in the MNT and less tissue damage compared to hot-iron disbudding, but to a stronger decrease and more tissue damage compared to the saline as control. Additionally, we expected a stronger decrease in the MNT after injection of clove oil compared with isoeugenol.

## 2. Materials and Methods

### 2.1. Animals and Housing

The study took place between August 2018 and June 2019 at the dairy facility of the University of Veterinary Medicine, Vienna at Kremesberg in Pottenstein, Austria. We included 40 calves (13 female and 27 male, 38 Simmental and two Simmental x Brown Swiss crosses, of which one was female and one male) that were born at the dairy facility between August 2018 and April 2019. Within the first hours of life, the calves were separated from the dam, bottle-fed with colostrum (at least 2 L) and brought to the outdoor calf hutches. As a standard procedure, all calves were injected with vitamins and ear-tagged within the first day of life. Calves stayed single-housed in outdoor hutches (147 cm × 109 cm × 117 cm) for the first 10 to 14 days; each hutch adjoined a fenced area (1.68 m²), where a hayrack and a water bucket were attached for ad libitum access. The hutches and the adjacent areas were bedded with straw. They were located next to each other under a roof for weather protection, allowing visual, auditory and limited tactile contact with conspecifics. In the first 5 days of life, the calves were fed with colostrum (2 L) three times daily (around 08:00 h, 13:00 h and 18:00 h), and afterwards with whole milk (3 L) twice daily. At 10 to 14 days of age, the calves were moved to groups in an open-fronted barn with deep litter and an elevated feeding area. They were grouped according to age with a maximum group size of nine and fed with whole milk (4 L) twice daily and hay and silage (depending on age) ad libitum. After 12 weeks of age, female calves were weaned of the milk. At the end of the experiment they stayed at the University’s farm or were sold to other dairy farms, one female calf was sold for slaughter. Male calves were fed with whole milk, hay and silage until they left the farm for fattening on another farm.

### 2.2. Study Design and Procedures

All procedures were discussed and approved by the institutional ethics and animal welfare committee (Vetmeduni Vienna) and the Advisory Committee for Animal Experiments of the Federal Ministry of Science, Research and Economics in accordance with GSP guidelines and national legislation (BMWFW-68.205/0049-WF/V/3b/16, date of approval: 31 March 2016).

**Figure 1:**
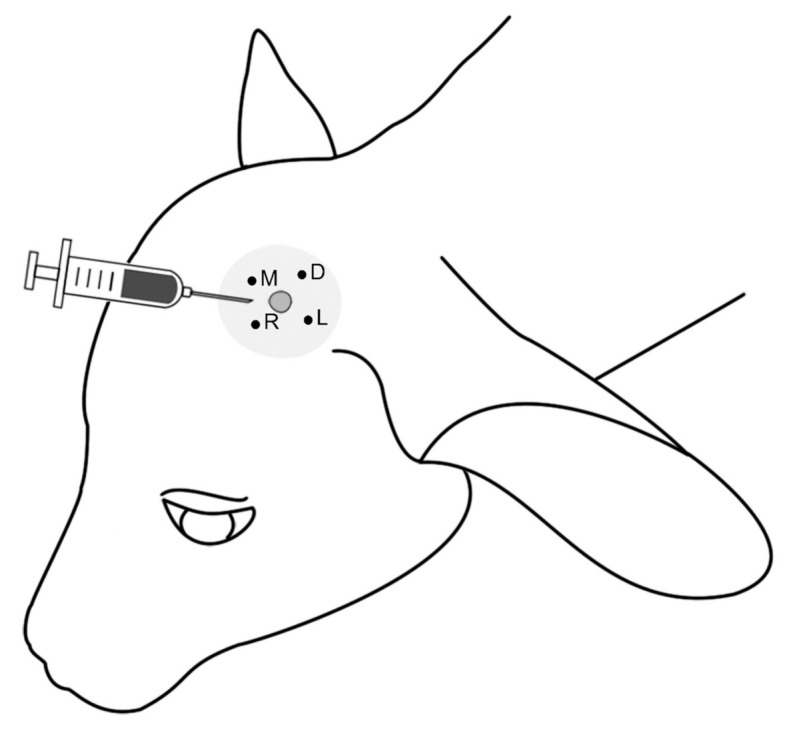
Locations used for the assessment of the mechanical nociceptive threshold (R: rostral, M: medial, L: lateral, D: dorsal). All four locations were used with the pressure algometer, only the lateral one with the von Frey filaments. The syringe indicates the direction of the saline, clove oil or isoeugenol injection.

The 40 calves were allocated randomly, by drawing notes, to four treatments (10 calves per treatment) at the age of 1 to 5 days: injection of 1.5 mL physiological saline (control, CON), of 1.5 mL clove oil (*Syzygium aromaticum*; 80.18% eugenol, Herba Chemosan Apotheker-AG, Austria; CLOV) or of 1.5 mL isoeugenol (99% isoeugenol, Merck KGaA, Germany; ISO) under each horn bud or hot-iron disbudding (BURN, with sedation and local anesthesia). For injection of saline, clove oil or isoeugenol, a 16 G needle (BOVIVET 16 G × 1 1/2” 1.6 × 38 mm, Jørgen KRUUSE A/S, Langeskov, Denmark) was inserted under the horn bud from rostro-medial of the bud in the direction of the base of the ear (Figure 1). Besides injection, no other procedure was applied to the horn bud, i.e., the horn bud was not removed. Calves disbudded with a hot iron were sedated with Xylazin (0.1 mg/kg, i.m., Sedaxylan 20 mg/mL, Eurovet Animal Health B.V., AE Bladel, Netherlands) and received a local block of the corneal nerve by injecting Procainhydrochlorid (5 mL s.c. each side; Procamidor 20 mg/mL, Richter Pharma AG, Wels, Austria) in the depression between the lateral canthus of the eye and the horn bud. Five minutes later the effectiveness of the local anesthesia was checked with a needle prick. We used the method of bud-off disbudding, removing the horn bud completely. Afterwards Cyclo spray (Chlorinetetracyclin Hydrochloride, Eurovet Animal Health BV, Bladel, Netherlands) was applied to the burns.

The treatments and all measurements took place in the home environment of the calves, i.e., in the single hutches or the group pens, depending on age. In general, one calf was treated per day; on 10 days, two calves were treated. Before the treatment and the first baseline measurements, the area around the horn buds was clipped and four different locations at a distance of 5 mm to the horn bud were marked using a permanent marker (Figure 1). The treatment was performed by AS or JS and took place at around 10:00 h, thus about 2 h after the morning feeding. During the injection, the calves were restrained on the ground by one or two persons; the head was pressed to the ground to allow for precise injection. The sedated calves in BURN were lying on their side without being restrained. If local reactions to the treatment occurred or in case of sickness, appropriate veterinary treatment was applied immediately as described in detail in the results. Swellings of the upper eyelids and suppurations after injection of clove oil (see results) occurred at a higher rate than would have been expected from previous studies [24,25]. Thus, we changed the bottle of clove oil after the treatments of the first three animals, as clove oil is a natural product without defined composition and we suspected that the composition of that specific batch might have caused these adverse effects.

### 2.3. Data Collection

All measurements and observations reported in this study were performed by one trained person (AJ) who was blind to the treatment, except for the BURN group where blinding was not possible.

#### 2.3.1. Mechanical Nociceptive Threshold

**Figure 2:**
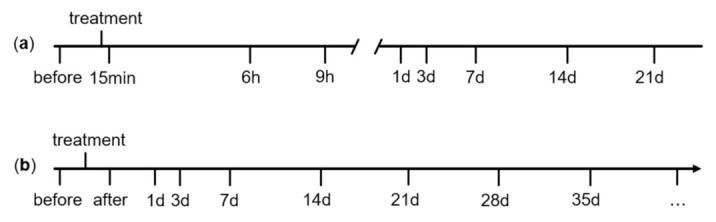
Time points of the measurement of the mechanical nociceptive threshold (**a**) and horn growth, wound healing and local tissue alterations (**b**). The latter measurements were repeated once weekly as long as the calf was at the farm.

The MNT was measured at the time points 1 h before (baseline, before) and 15 min, 6 h, 9 h, 1 d, 3 d, 7 d, 14 d, and 21 d after the treatment (Figure 2A), using von Frey filaments (Aesthesio, Ugobasile SRL, Gemonio, Italy) and a pressure algometer (ProdPlus, Topcat Metrology Ltd., Downham Common, Ely, Cambridgeshire, UK). With vFF, lower pressures between 0.08 mN and 2.94 N (corresponding to a theoretical pressure of 2.53–292 g/mm^2^) can be assessed, with the PA pressures between 0.5 and 20 N (corresponding to a theoretical pressure of 50–2040 g/mm^2^). We always started measuring the MNT with the vFF, only at the lateral location on both sides, followed by measuring with the PA at all four locations (Figure 1). The measurements were conducted either without restraining the calves as long as the calves were single housed, or with gentle restraint (holding the animal at the front chest/neck and back to prevent them from moving away, the head not being restrained) by a second person at time points 14 d and 21 d when the calves were group housed. As the MNT measurements of the group-housed animals needed more time and additional staff, it was not possible to extend the period of measurement past day 21 after the treatment. We defined a withdrawal response as a clear head movement away from the probe (filament or metal tip) due to the stimulus. To ensure that the movement was caused by the stimulus, the measurement only started when the calf was calmly standing or lying, its head was not moving and no disturbances (e.g., flies at the head) were present. Additionally, the probe was applied slowly to the skin, touching the skin for 2 s (touch phase) before applying pressure. If the calf showed no reaction during the touch phase, pressure was applied.

When applying the vFF, we used the up-and-down method described by McMackin et al. [26], always starting with the thinnest filament and applying the next thicker filaments increasing force, until a clear withdrawal response was visible. Then, we lowered the force by two filaments and started to increase the force again until the calf withdrew again and repeated the procedure another time, recording three values in total. The measurement always started on the left side.

For the measurements with the PA, we used a 2 mm metal tip and increased the force applied at a rate of 2 N/s until the calf withdrew its head; the increase in force was indicated by a green light if below, by no light if equal to or by a red light if above 2 N/s. The PA measurements were taken in the order rostral, medial, lateral and dorsal, always starting with the left side and alternating between sides per location—rostral left, rostral right, medial left, medial right,, etc. If the calf showed a withdrawal response during the 2-s touch phase the probe was removed, the experimenter waited until the calf’s head was at rest again and then restarted with putting the probe on the skin. This was repeated a maximum of three times for each measurement point; in case there was a withdrawal response during the touch phase all three times, we assigned a value of 0 to this measurement. Only one measurement per location was performed to reduce the risk of habituation [27] and the duration of interacting with the animal.

#### 2.3.2. Horn Growth and Tissue Alterations Including Wound Healing

For simplicity, we will refer to the growth and height of both horn and bud as horn growth and horn height. We measured the horn height using a hollow cylinder with a plunger (Supplementary Figure S1), with a measuring range from 0 to 20 mm. The hollow cylinder was handmade and validated by measuring screw nuts of various known sizes. If the horn height exceeded 20 mm, we used a slide gauge. It was also used to determine the size of the burn wounds and other pathological alterations of the area around the horn buds. We defined the presence of horn growth as an increase in height of more than 2 mm, compared to the horn height before the treatment. As the wounds were usually not completely round, the wound surface was calculated by measuring the length and width of the wound and using the formula for an ellipse. Wounds were defined as healed if only a scar was visible. Additionally, we examined the area around the horn buds and the eyelids by adspection and palpation and assessed the occurrence of the following tissue alterations: desquamation, discoloration, exudation, granulation, incrustation, necrotic-like tissue, tissue retraction, suppuration and swellings. A precise description with definitions modified from Baungartner [28] or Anonymous [29] is given in Supplementary Table S1. To investigate if the altered tissue was painful, we applied slight pressure with the fingertip. Due to limited space, we will only report the main findings in the results. The parameters were measured before and after the treatment, as well as 1 d, 3 d and 7 d later, and then at a weekly interval (Figure 2B) as long as the calf was at the farm, i.e., 7–28 weeks, on average 13.5 ± 0.75 weeks. In this paper, we only report findings until week 12 as at least half of the calves of each treatment were still at the farm.

### 2.4. Statistical Analysis

All statistical analyses were performed using SPSS (Version 25.0, IBM Corp., Armonk, NY, USA). For MNT and horn growth, we used linear mixed models (LMM) with the fixed effects treatment, time point, sex, their three-way interaction and all lower-order interactions; we included age at treatment as a covariate and the animal as a random effect. As dependent variables, we used the mean of all measures for vFF (six values) and for PA (eight values). We used Bonferroni corrections for post hoc comparisons. Regarding the horn growth, we added the head side as a fixed effect, used log-transformed (log10) data and only the time points before, 14 d, 28 d, 42 d, 56 d, 70 d and 84 d for the analysis, as at least five calves per treatment (CON = 5, CLOV = 6, ISO = 5) had been at the farm at 84 d after the treatment. The sample size regarding the horn height varies due to missing values (failed measurement or inability to measure due to swellings of the horn bud region). Models were reduced by eliminating non-significant (*p* > 0.5) factors starting with the three-way interactions, followed by the two-way interactions and, finally, the main effects, except for the variables of main interest: time point, treatment and their interaction. In the discussion, we do not address significant effects of confounding factors (sex, age, head side), as they were outside the scope of the paper. Model assumptions (normal distribution and homogeneity of variance of residuals) were checked graphically. For the tissue alterations and wound healing, descriptive statistics are presented. For all statistical tests, an alpha level of 0.05 was set for significance.

## 3. Results

### 3.1. Mechanical Nociceptive Threshold

#### 3.1.1. Von Frey Filaments

There was an effect of treatment depending on time (interaction treatment*time, F_23,49_ = 2.97, *p* = 0.001), as well as a main effect of both treatment (F_3,32_ = 6.63, *p* = 0.001) and time (F_8,45_ = 12.26, *p* < 0.001) on the MNT (complete model results including the confounding factors in Supplementary Table S2).

**Figure 3:**
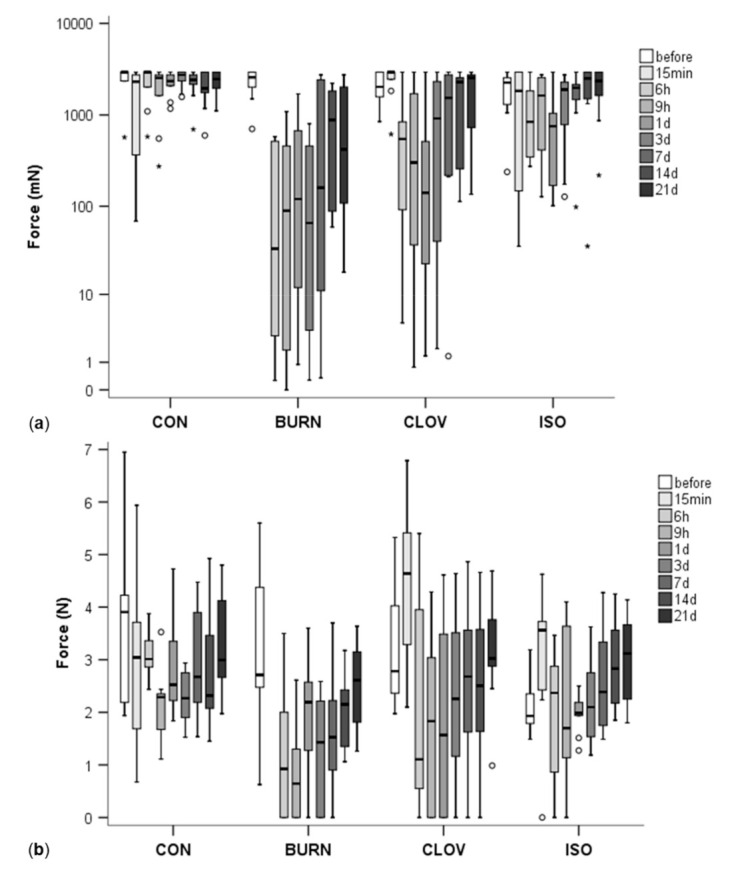
Mechanical nociceptive threshold (MNT) measured with von Frey filaments (applied force in mN, (**a**), logarithmic scale) or a pressure algometer (applied force in N, (**b**)) in the four different treatments—injection of saline (CON), clove oil (CLOV), isoeugenol (ISO) or disbudding with a hot iron (BURN). The boxplots represent the mean MNT across the different locations of measurement at different time points. No measurement exists for BURN at 15 min due to sedation. The box-and-whisker-plots show the median (line in box), 25 and 75% quartile (bottom and top end of the box) and minimum and maximum (whiskers) except for outliers (circles, distance to box 1.5–3 times the interquartile range) and extreme values (asterisks, distance to box > 3 times the interquartile range).

BURN calves showed the strongest decrease of the MNT, and consequently the highest sensitivity, over all time points, with values remaining lower than at the beginning up to day 21 (Figure 3a, Table 1; no value at 15 min due to sedation) and differing from CON significantly or by trend up to day 21 (*p* < 0.001 for 6 h, 9 h, 1 d, 3 d; *p* = 0.027 for 7 d; *p* = 0.09 for 21 d). The second strongest decrease of the MNT was observed in CLOV and lasted up to day 3, differing from CON at 6 h (*p* = 0.004), 9 h (*p* = 0.041) and days 1 and 3 (*p* < 0.001). However, 15 min after the injection the MNT was slightly increased, then it decreased, with the lowest values at 1 d after the injection, and increased thereafter with high inter-individual variation (Figure 3a). In some calves, the decrease of the MNT lasted until 21 days after the treatment. In ISO the decrease of the MNT was even weaker; it differed from CON only at the two time points 1 d (*p* < 0.001) and, by trend, 3 d (*p* = 0.077, Table 1). In contrast to CLOV, the MNT decreased immediately after the treatment in most ISO calves (although not significantly); the lowest values were reached again 1 d after injection, but values returned to baseline faster than in CLOV. In CON calves, the MNT stayed highest with only a small, short-term, non-significant decrease 15 min after injection. There was no significant difference between CLOV and BURN or CLOV and ISO at any time point, but ISO calves had a higher MNT than BURN at 6 h (*p* = 0.053), 9 h (*p* = 0.011) and 1 d (*p* = 0.001, Table 1).

#### 3.1.2. Pressure Algometer

There was an interaction of treatment and time (F_23,50_ = 2.01, *p* = 0.020), as well as a main effect of time (F_8,50_ = 4.13, *p* = 0.001), but no main effect of treatment (F_3,26_ = 2.07, *p* = 0.129). Supplementary Table S2 contains the complete model results including confounding factors.

The time courses of the MNT measured by PA were similar to those measured with vFF: BURN calves showed the longest and strongest decrease of the MNT, followed by CLOV, then ISO and finally CON, although the differences were less clear and often not statistically significant in post hoc tests with Bonferroni correction (Figure 3b, Table 1). Again, there was an increase of the MNT in CLOV calves 15 min after the treatment as compared to baseline, with values significantly higher than in CON (*p* = 0.045, Table 1), which was found here also for the ISO calves. Thereafter the MNT decreased in both CLOV and ISO, with the lowest values at 1 d (CLOV) or 6 h and 1 d (ISO), and went back to the basal level within the first 3 days after injection in ISO, whereas in CLOV, this took again 3 weeks (Table 1). Several calves in BURN and CLOV and one ISO calf showed a withdrawal reaction at the slightest touch of the skin with the PA equal to a value of 0, which never happened in CON calves (Figure 3b). The MNT in CON also decreased from baseline after injection, with the lowest value 9 h later, differing from before (*p* = 0.036), and stayed somewhat lower than baseline on average (Table 1). However, large decreases as in the other treatments did not occur (Figure 3b, Table 1). Nevertheless, CON had a significantly higher MNT compared to BURN only at 6 h (*p* = 0.023) and by trend to CLOV at 1 d (*p* = 0.056, Table 1).

### 3.2. Horn Growth and Tissue Reactions Including Wound Healing

#### 3.2.1. Horn Growth

**Figure 4:**
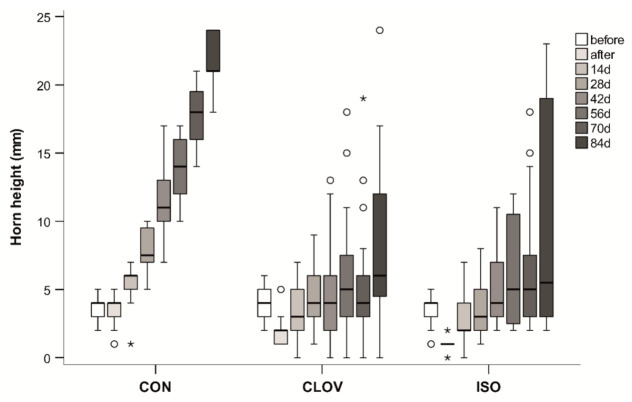
Horn height (in mm) of the calves in the three injection groups—saline (CON), clove oil (CLOV) and isoeugenol (ISO)—shown for different time points until week 12. The decrease at time point after in CLOV and ISO is due to swellings around the horn buds after the injection. The hot-iron treatment is not included, as no horn growth occurred. The maximum sample size was 20 horns for all treatments (2 horns/animal) at each time point; due to missing data the actual sample size was as follows: CON: 14/18/20/20/20/18/16/10; CLOV: 16/14/17/13/18/19/17/12; ISO: 16/12/20/20/19/20/16/10. The box-and-whisker-plots show the median (line in box), 25 and 75% quartile (bottom and top end of the box) and minimum and maximum (whiskers) except for outliers (circles, distance to box 1.5–3 times the interquartile range) and extreme values (asterisks, distance to box > 3 times the interquartile range).

No horn growth occurred in BURN calves as long as the calves were at the farm (i.e., up to 11–24 weeks). Therefore, we excluded BURN calves from further analysis. There was an interaction of treatment and time point (F_12,58_ = 17.45, *p* < 0.001, Figure 4), as well as a main effect of treatment (F_2,26_ = 13.23, *p* < 0.001), time point (F_6,57_ = 65.03, *p* < 0.001) and sex (F_1,24_ = 4.44, *p* = 0.046). In the first 12 weeks the horns grew continuously in CON calves, while growth stopped, was delayed or similar to CON in CLOV and ISO calves (Figure 4, Supplementary Table S3). There was no significant difference between CLOV and ISO treatment (*p* > 0.99).

Twelve weeks after the treatment, the mean horn height in the animals still at the farm was 21.5 ± 2.07 mm (mean ± SD) in CON (minimum-maximum: 18–24 mm, *n* = 5 animals, 10 horns), 8.7 ± 6.76 mm in CLOV (0–24 mm, 12 horns) and 10.0 ± 8.58 mm in ISO (2–23 mm, 10 horns). Growth was absent in six of 12 horns in CLOV and five of 10 horns in ISO. However, the treatment effectively disrupted horn growth on both sides of the head only in one calf in CLOV and in ISO. In both treatments, we actually observed a decrease in horn height after the treatment compared to before the treatment for about 5 weeks. It was caused by swellings as well as retraction of the tissue including the horn buds. As the diameter of the hollow cylinder was bigger than the area of the tissue retraction, it was not possible to assess the full horn height; these data were excluded from analysis.

In CLOV and ISO calves, we found changes of the shape of the horns (e.g., depressions on different parts of the horn), as well as scurs (a horn that is not attached to the skull; one horn in CLOV) and the splitting of the horn in two parts (two horns in CLOV, one horn in ISO); in CON, all horns were shaped normally.

#### 3.2.2. Wound Healing and Tissue Alterations

After hot-iron disbudding, the wound surface was on average 285.5 ± 50.49 mm² (mean ± SD). Disbudding wounds needed 5.3 ± 0.95 weeks to heal (minimum: 3, maximum: 7). The most common type of tissue alterations in BURN calves were incrustations, which were observed in all animals on both sides 2 weeks after disbudding, followed by exudation and granulation. Additionally, we observed mild to moderate swelling of the tissue around the wound in all calves for up to 1 week after disbudding. We did not detect any signs of suppuration in BURN calves during our study. We refrain from a more detailed description of the course of wound healing in BURN calves, as a comparable one has been provided in previous studies [5,30].

Injection of saline did not lead to any tissue alterations in any of the calves. Immediately after injection of clove oil and isoeugenol, the area around the horn bud (*n* = 20 horn bud areas/treatment, two per animal) was swollen in all animals on both sides lasting at least for 1 day up to 2 (ISO) or 3 (CLOV) weeks (Table **2**). On the day after the treatment, mild to severe swellings occurred in all CLOV calves on both upper eyelids (four mild, four moderate and 12 severe), while swellings occurred rarely in ISO calves (three calves with only one eyelid swollen; two mild and one moderate swelling, Supplementary Figure S2). One of the CLOV calves with severe swelling of the upper eyelids developed the same condition at one lower eyelid. Due to the severe swelling of the eyelid, which and impaired sight, four CLOV calves were not able to find their milk bucket without assistance in the morning after the treatment. After the swelling was diagnosed and the MNT measured, nine calves from CLOV were treated with a single injection of 2–5 mL Dexa “Vana” (2 mg/mL Dexamethasone Dinatrium Phosphate, Vana GmbH, Vienna, Austria) and one calf with two injections of 3 mL Rifen (100 mg/mL Ketoprofen, Richter Pharma AG, Wels, Austria). The therapy led to a reduction of the swellings within a few hours. As the swelling of the calves’ eyelids in ISO were only mild or moderate, no treatment was necessary. Discolorations (mostly black) occurred also frequently within hours after injection; their number decreased clearly only from week 5 onwards (Table 2). Necrotic-like tissue type I occurred mainly from week 2 to week 5, in six ISO calves and nine CLOV calves (Table 2). Incrustations were observed in seven ISO and nine CLOV calves, while retraction of tissue was found in all animals except one in both treatments (CLOV/ISO), persisting in half of the animals until week 11 (Table 2). Necrotic-like tissue type II occurred in six CLOV calves (Table 2).

Two to 4 weeks (3 ± 0.31 weeks) after the treatment, suppurations occurred at the injection sites in six CLOV calves and two ISO calves (Table 2, Supplementary Figure S2). Every 2–3 days, the infected sites were cleaned using a diluted solution of Betaisodona (Povidon-Iod, Mundipharma GmbH, Frankfurt am Main, Germany) and gauze swabs (ES gauze swabs, 17 threads, 8-ply, 10 × 10 cm, Hartmann group, Heidenheim, Germany). After cleaning the wounds, Cyclo spray was applied. On average, suppurations occurred for 2.06 ± 1.6 weeks (minimum: 1, maximum: 5). One calf with suppurations (CLOV) developed fever and reduced appetite and was additionally treated with Roxilin (172 mg/mL Amoxicillin Trihydrat, Richter Pharma AG, Wels, Austria); this calf developed a bone sequestration of the skull under the horn bud that was diagnosed by computer tomography.

## 4. Discussion

### 4.1. Mechanical Nociceptive Threshold

We investigated the effect of clove oil and isoeugenol on the MNT for up to 3 weeks after the treatment. The results support our hypothesis that injection of clove oil and, especially, isoeugenol lead to a lower sensitivity as compared to hot-iron disbudding without analgesia, but higher sensitivity compared to injection of saline. As predicted, isoeugenol increased the sensitivity to a lower extent and for a shorter time than the injection of clove oil.

The slightly non-significantly heightened sensitivity that occurred in CON 15 min after the injection might have been caused by the needle prick [31] and/or by heightened pressure on the tissue caused by the administration of the liquid [32]. Six hours after the injection, the MNT measured with vFF in CON returned to baseline. In contrast, the median MNT measured with the PA dropped further until 9 h before rising again. This might be due to the different measurement ranges of the devices, as the median was slightly above 3 N (PA), which is higher than the maximum cut-off of the vFF, in combination with the different locations of measurements. Further, we cannot completely rule out that a slight general sensitization might have occurred for the use of the PA, as previously described by Janczak et al. [27].

The injection of both clove oil and, to a lower extent, isoeugenol led to a lower MNT and thus a higher sensitivity compared to the injection of saline. The skin sensitizing potential of clove oil and eugenol [33], and isoeugenol [34] and/or their cytotoxic properties [12,14] might have led to the heightened sensitivity. Prashar et al. [22] suppose that the cytotoxic effect of clove oil or eugenol damages the cell membranes, which leads to necrosis of the tissue and provokes pain [35]. The increased MNT 15 min after the treatment in CLOV as compared to CON might have been caused by their anesthetic properties [fish: 23, rats: 36]. This effect occurred also in a previous study on calves and goat kids [24]. For ISO, we found an anesthetic effect only with the PA (although not significant after Bonferroni correction) but not with the vFF; this might be due to the different locations used for the assessment; they may differ in general sensitivity, or the liquids might not have dispersed evenly [10,24]. Six hours after the treatment, the sensitivity increased strongly in CLOV calves and to a lower extent in ISO calves, which indicates that the anesthetic effect of these substances is only temporary. When using clove oil as a topical anesthetic on the cornea of rats (200 μg/eye), the anesthetic effect only lasted for 15 min [36], which corresponds to our findings.

In ISO calves, the increase in sensitivity was lower and, notably, lasted for a shorter time than in CLOV calves, probably due to the fact that clove oil contains additional active substances [12], which are known to possess sensitizing properties [33]. CLOV and ISO also differed with regard to indications of hyperalgesia and allodynia. Hyperalgesia is an increased pain response to a stimulus that under normal conditions is painful, whereas allodynia is defined as a pain response to a stimulus that normally does not cause pain [37]. In some ISO calves and in all CLOV and BURN calves, signs of hyperalgesia (clearly enhanced sensitivity) were common, whereas signs of allodynia (reactions at the slightest touch with the PA, without applying pressure, and reactions to the low evaluator sizes of the vFF) mainly occurred in CLOV and BURN calves. The duration of signs of hyperalgesia was shorter in ISO calves compared to CLOV and longest in BURN calves, where they existed up to 3 weeks after treatment. The heightened sensitivity of CLOV on days 1 and 3, similar to BURN, is in accordance with Sutherland et al. [10]: they reported a heightened sensitivity 48 h after injection of clove oil, which was similar to the sensitivity caused by hot-iron disbudding.

The major increase of the MNT 6 h after the treatment in BURN reflects the acute pain associated with the use of a hot iron without post-treatment analgesia, which has already been shown and discussed as a major welfare issue in the literature [3,7,9]. The occurrence of hyperalgesia, as reflected in the lowered MNT, up to 3 weeks after disbudding confirms previous findings [5,6] and suggests that calves experience ongoing pain during this time [38]. In addition, the prolonged period of hyperalgesia may constitute a high risk for the development of a long-lasting increase in pain sensitivity or chronic pain (in humans: Kaasa et al. [39]), further enhanced by the young age of the calves: the risk for developing increased pain sensitivity after a painful procedure is higher in neonates as compared to animals at an older age (e.g., [5,40]). Thus, the risk for long-term effects on pain sensitivity likely is highest for hot-iron disbudded calves due to the longest and strongest hyperalgesia, followed by clove oil injection; despite the relatively short duration of hyperalgesia, it can neither be excluded after isoeugenol injection, due to the young age of the animals.

Differences between treatments were confirmed in particular when using the vFF, pointing at the higher sensitivity of these measurement devices possibly due to the lower forces that could be applied, as indicated already in a previous study [24]. When using a pressure algometer, Reedman et al. [41] could not detect any difference in MNT between sham-disbudded calves and calves disbudded with caustic paste with or without use of local anesthetics and/or meloxicam from 3 h until 7 d after the treatment. Besides the measurement range per se, the different characteristics of the probe (e.g., material, size) may be relevant for differences between PA and vFF. There is evidence for a difference in activated nerve fibres: while vFF stimulate A-delta and A-beta fibres but not C fibres, pressure algometers stimulate A-delta and C fibres [42,43]. Further, different locations of measurement might also have contributed to the different sensitivities of vFF and PA in our study.

By measuring MNT we only assess pain provoked by a stimulus. Previous studies indicate that such increased pain sensitivity around hot-iron wounds comes along with signs of spontaneous pain as well as with systemic changes in pain sensitivity with potential negative effects on welfare [5,6,38]. After clove oil injection calves similarly exhibit both increased pain sensitivity measured by MNT and behavioral signs of (spontaneous) pain within the first 48 h after the treatment [10]. It remains unclear how long calves experience spontaneous pain after clove oil or isoeugenol injection, although MNT results together with previous studies and results on tissue alterations (see below) suggest some lasting effects.

### 4.2. Horn Growth

Twelve weeks after the treatment, at least one of two horn buds was growing in 84% and 80% of the calves after injection of clove oil and isoeugenol, respectively, although the horn growth was on average lower than in the control treatment. However, we cannot exclude that horn growth was just delayed. In a similar study, no horns and only 13% scurs were observed in calves 6 months after treatment with clove oil [16], whereas 10 months later, 5% horns and 63% scurs were observed in the same animals [17]. We found a similar pattern in two calves that did not show horn growth 21 weeks after injection in a preliminary study [25], but had to be dehorned 1 year later. In sum, there are strong indications that clove oil rather delays than fully prevents horn growth. Additionally, in our study with a limited number of calves increasing the dose from 0.5 mL/horn bud [10,15,16] to 1.5 mL/horn bud, which seemed promising according to a preliminary study [25], did not improve the outcome. However, further studies on a larger number of animals are required to investigate the potential effects of different dosage and application techniques, especially regarding the use of isoeugenol.

An insufficient concentration of eugenol in the clove oil or breed differences might be further reasons why in our study horn growth could be prevented only in half of the horns in CLOV and ISO calves in contrast to other studies using clove oil [15,16]. The concentration of eugenol in clove oil can vary from 47.64% [44] up to 88.58% [12]. Thus the concentration in the clove oil we used (80.18%) might have been lower than in the other studies; as the other studies did not report the concentration, we cannot make any comparisons. Sutherland et al. [10,16] worked with Holstein Friesians, whereas our calves were Simmental cattle; the breeds differ in horn and skull morphology [45] and thus likely also in the size of horn bud tissues. Goat kids have more developed horn buds as compared to calves and the effectiveness of an injection of clove oil was much lower (13% after 5 months, Hempstead et al. [21]) as compared to Holstein Friesian calves (87% after 6 months, Sutherland et al. [16]). However, this does not apply to BURN, as we completely removed the horn bud area including the horn-forming cells; the bud-off method was also the most effective method used in calves by Sutherland et al. [16] and in goats by Hempstead et al. [21].

### 4.3. Wound Healing and Tissue Alterations

Hot-iron disbudding leads to open wounds with a healing duration of several weeks (7 to 10 weeks until wounds were re-epithelialized), which poses a high risk for infections and additional pain [5,6,16]. Disbudding wounds remain sensitive until the wounds are completely healed [5] or even longer [6]. In our study it took on average 5 weeks until the BURN wounds healed, thus less time compared to other studies. After injection of clove oil or isoeugenol, we did not observe any open wounds like in the BURN calves, though we observed various other tissue alterations (Table 2, Supplementary Table S1).

We observed mild to severe swellings of the upper eyelids in all CLOV calves, while only two mild and one moderate swelling of an eyelid occurred in ISO calves (Supplementary Figure S2). Clove oil, eugenol [33] and isoeugenol [34] have sensitizing properties causing an allergic reaction [46,47] and therefore might have led to these swellings. Most of the other observed alterations (discoloration, tissue retraction, necrotic-like tissue type I and II) were probably a sign of necrosis [22]. Six CLOV and two ISO calves additionally developed suppurations at the injection site 2 to 4 weeks after injection (CLOV twice on one side and four times on both sides, ISO both on one side). This might have been caused by the cytotoxic effect of the injectant [12,22] that is necessary for disbudding. The anti-inflammatory properties of the injectants might have led to a delay in the appearance of the suppurations [12,13]. Furthermore, the closure of the injections site was delayed compared to the control (personal observation of AJ), which might have constituted an entry point for infectious agents. Sutherland et al. [16] and Hempstead et al. [21] also reported signs of infection after injection of clove oil, but did not report on the extent.

One CLOV calf had to be treated with antibiotics due to fever and reduced appetite, associated with severe suppurations for several weeks and a bone sequestration of the skull. Thus, the use of clove oil can lead to tissue damage of comparable severity to damage after hot-iron disbudding. However, the risk for such severe tissue damage is still higher when using a hot iron, as indicated by computer tomography images of our calves, where skull bone destruction was found in all five investigated BURN calves [48].

The high occurrence of tissue alterations in CLOV may be dose-dependent. In previous studies of our working group [24,25], tissue alterations were observed quite rarely. The injected volume was three times lower (0.5 mL) in Frahm et al. [24] compared to our 1.5 mL per horn bud. Similarly, the volume was lower for most calves in Schoiswohl et al. [25] (only two calves received 1.5 mL, two 1 mL and two 0.5 mL).

In comparison with CLOV, the number of animals affected and the severity of tissue alterations were far lower in ISO calves. This again may relate to the composition of clove oil including several constituents besides eugenol that can induce hypersensitivity [22], in contrast to the pure substance isoeugenol.

## 5. Conclusions

Regarding pain sensitivity, i.e., mechanical nociceptive threshold, and tissue alterations, the use of isoeugenol is preferable compared to clove oil or hot-iron disbudding without analgesia. While clove oil still induces somewhat lower pain compared to hot-iron disbudding, its use for disbudding cannot be recommended due to the risk of tissue alterations and the inadequate prevention of horn growth. Although the effectiveness of isoeugenol to prevent horn growth was equally insufficient, the much shorter and weaker effects on pain sensitivity indicate benefits for calf welfare as compared to hot-iron disbudding and thus merit further research focusing on enhancing effectiveness, for example via improved administration techniques. Isoeugenol may be an alternative to hot-iron disbudding only if sufficiently effective. Since the injection of isoeugenol also induces pain, the effectiveness of analgesic drugs needs to be investigated as well.

## Figures and Tables

**Table 1:** Mechanical nociceptive threshold measured with von Frey filaments (vFF) or a pressure algometer (PA) for the four treatments—injection of saline (CON), clove oil (CLOV) or isoeugenol (ISO) and hot-iron disbudding (BURN). Data are presented over all time points (treatment) or for each time point, i.e., before treatment or the indicated minutes, hours or days after treatment depicted by the interaction treatment*time point. Values show estimated means ± standard error of the force applied. *n* = 10 calves/treatment.

Factors	CON	BURN	CLOV	ISO
VFF (mN)				
Treatment	2147 ± 178 ^a^	808 ± 172 ^c^	1309 ± 181 ^bc^	1664 ± 171 ^ab^
Treatment*time point				
Before	2480 ± 277	2363 ± 273 ^x^	1980 ± 279 ^x^	1948 ± 273 ^x^
15 min	1752 ± 331	-	2345 ± 333 ^x^	1670 ± 328
6 h	2170 ± 281 ^a^	221 ± 265 ^c,y^	759 ± 271 ^bc,y^	1243 ± 265 ^ab^
9 h	1901 ± 280 ^a^	289 ± 264 ^c,y^	804 ± 270 ^bc,y^	1518 ± 264 ^ab^
1 d	2173 ± 225 ^a^	413 ± 220 ^b,y^	552 ± 227 ^b,y^	911 ± 220 ^b,y^
3 d	2476 ± 237 ^a^	256 ± 233 ^c,y^	940 ± 262 ^bc,y^	1618 ± 233 ^ab^
7 d	2129 ± 301 ^a^	891 ± 285 ^b,y^	1327 ± 291 ^ab^	1811 ± 285 ^ab^
14 d	1985 ± 340	995 ± 337 ^y^	1491 ± 341	2170 ± 337 ^x^
21 d	2254 ± 341	1036 ± 338 ^y^	1584 ± 359	2090 ± 338 ^x^
PA (N)				
Treatment	2.93 ± 0.253 ^a^	1.87 ± 0.232 ^b^	2.54 ± 0.249 ^ab^	2.48 ± 0.237 ^ab^
Treatment*time point				
Before	4.01 ± 0.513 ^xy^	3.17 ± 0.481 ^x^	3.17 ± 0.503	2.22 ± 0.493
15 min	2.95 ± 0.453 ^a^	-	4.51 ± 0.437 ^b,x^	3.12 ± 0.428 ^ab^
6 h	2.85 ± 0.399 ^a^	1.23 ± 0.356 ^b,y^	1.85 ± 0.375 ^ab,z^	1.86 ± 0.364 ^ab,x^
9 h	2.02 ± 0.412 ^z^	0.86 ± 0.368 ^y^	1.69 ± 0.387 ^z^	2.14 ± 0.377
1 d	2.81 ± 0.341 ^a^	2.09 ± 0.317 ^ab^	1.56 ± 0.335 ^b,z^	1.95 ± 0.325 ^ab,x^
3 d	2.29 ± 0.322 ^yz^	1.28 ± 0.298 ^y^	2.03 ± 0.353 ^z^	2.22 ± 0.306
7 d	2.86 ± 0.370	1.70 ± 0.345	2.34 ± 0.364 ^yz^	2.55 ± 0.354
14 d	3.05 ± 0.383	2.06 ± 0.357	2.31 ± 0.376 ^yz^	3.05 ± 0.366
21 d	3.54 ± 0.356 ^x^	2.56 ± 0.331 ^x^	3.45 ± 0.372 ^xy^	3.24 ± 0.340 ^y^

^a,b,c^ Estimated means followed by a different letter differ significantly (*p* ≤ 0.05, after Bonferroni corrections, BC) in the row; ^x,y,z^ values in a column with different superscripts differ significantly (*p* ≤ 0.05 after BC).

**Table 2:** Prevalence of tissue alterations observed after injection of clove oil (CLOV) or isoeugenol (ISO) under the horn buds until week 11. Numbers of calves with the respective tissue alterations on one or both sides of the head (one/both) are presented. For detailed definitions, see Supplementary Table S1. No tissue alterations were observed after injection of saline. Wounds of calves disbudded with a hot iron are not presented due to a different type of tissue alteration (exclusively incrustations and swellings around the horn bud).

Tissue Alterations	Days after Treatment ^1^													
Treatment	0 ^2^	1	3	7	14	21	28	35	42	49	56	63	70	77
Discoloration														
CLOV	3/5	2/8	3/5	2/8	2/8	4/5	6/3	4/2	3/0	3/0	3/0	3/0	2/0	1/0
ISO	1/9	2/8	2/7	5/5	4/5	6/3	6/2	4/1	3/1	2/0	0/0	0/0	0/0	0/0
Incrustation														
CLOV	0/0	0/0	0/0	1/0	1/3	2/4	2/4	3/3	2/2	2/2	1/0	0/0	0/0	0/0
ISO	0/0	0/0	1/0	2/0	2/0	2/0	1/1	1/1	1/1	2/0	1/0	1/0	0/0	0/0
Necrotic-like tissue														
Type I														
CLOV	0/0	0/0	1/0	2/1	4/5	4/4	5/2	3/1	2/0	2/0	1/0	1/0	1/0	1/0
ISO	0/0	0/0	0/0	3/1	4/1	5/1	4/1	4/1	3/1	2/0	0/0	0/0	0/0	0/0
Type II **^3^**														
CLOV	0/0	0/0	0/0	0/0	1/0	1/1	3/1	4/2	3/0	1/0	1/0	1/0	1/0	0/0
Tissue retraction														
CLOV	0/0	1/1	1/5	2/6	2/8	4/6	4/5	2/5	2/5	2/5	2/5	2/5	1/5	1/4
ISO	0/0	1/1	1/5	2/7	0/9	1/8	1/8	1/8	0/8	1/7	1/7	2/4	2/4	2/3
Suppuration														
CLOV	0/0	0/0	0/0	1/0	2/2	2/3	2/3	2/2	1/0	1/0	0/0	0/0	0/0	0/0
ISO	0/0	0/0	0/0	0/0	0/0	0/0	2/0	1/0	1/0	0/0	0/0	0/0	0/0	0/0
Swelling around horn bud														
CLOV	/10	/10	0/6	0/1	1/0	1/0	0/0	0/0	0/0	0/0	0/0	0/0	0/0	0/0
ISO	/10	/10	3/5	1/1	1/0	0/0	0/0	0/0	0/0	0/0	0/0	0/0	0/0	0/0
Swelling of upper eyelid														
CLOV	1/1	/10	1/7	3/4	1/0	0/0	0/0	0/0	0/0	0/0	0/0	0/0	0/0	0/0
ISO	1/0	3/0	1/0	1/0	0/0	0/0	0/0	0/0	0/0	0/0	0/0	0/0	0/0	0/0

^1^ The sample size was *n* = 10 for all time points and treatments up to day 56, but varied thereafter: CLOV/ISO on day 63 10/8, day 70 9/8 and day 77 7/6. ^2^ Day 0 corresponds to the time point 15 min after the treatment. ^3^ No Type II found in ISO.

## Data Availability

The data presented in this study are available on request from the corresponding author.

## Supplementary Materials

The following are available online at https://www.mdpi.com/2076-2615/11/3/828/s1: Figure S1. Measurement devices used for the assessment of horn height and the size of the burn wounds and tissue alterations; Figure S2. Local reactions after injection of clove oil or isoeugenol; Table S1. Tissue alterations assessed during the clinical inspection and palpation of calves with photographs, definitions and scoring; Table S2. Results of the linear mixed model for the mechanical nociceptive threshold measured with von Frey filaments (vFF) and a pressure algometer (PA) in calves of the four different treatments (injection of saline, clove oil or iseugenol or the disbudding with a hot iron); Table S3. Horn height measured with a hollow cylinder and a slide gauge in calves of the three injection treatments—injection of saline (CON), clove oil (CLOV) or isoeugenol (ISO).
