# Supplementary material for: Mechanical Nociceptive Threshold, Tissue Alterations and Horn Growth in Calves after Injection of Isoeugenol or Clove Oil under the Horn Bud
Source: Animals (Basel). 2021 Mar 15;11(3):828. doi: 10.3390/ani11030828 (PMC8000045; doi:10.3390/ani11030828)
Supplement: Supplementary file 1 [file animals-11-00828-s001.pdf]

animals

MDPI

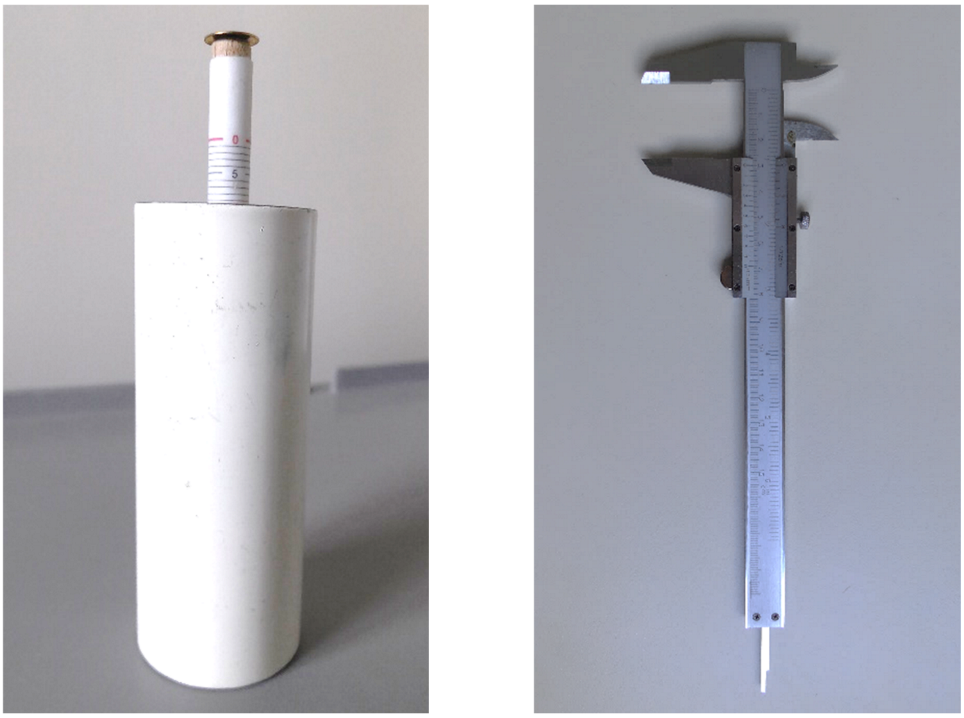

Figure S1. Measurement devices used for the assessment of horn height (both) and the size of the burn wounds and tissue alterations (only slide gauge). Left: handmade hollow cylinder with a plunger; measuring range 0–20 mm. Right: slide gauge, measuring range 0–160 mm.

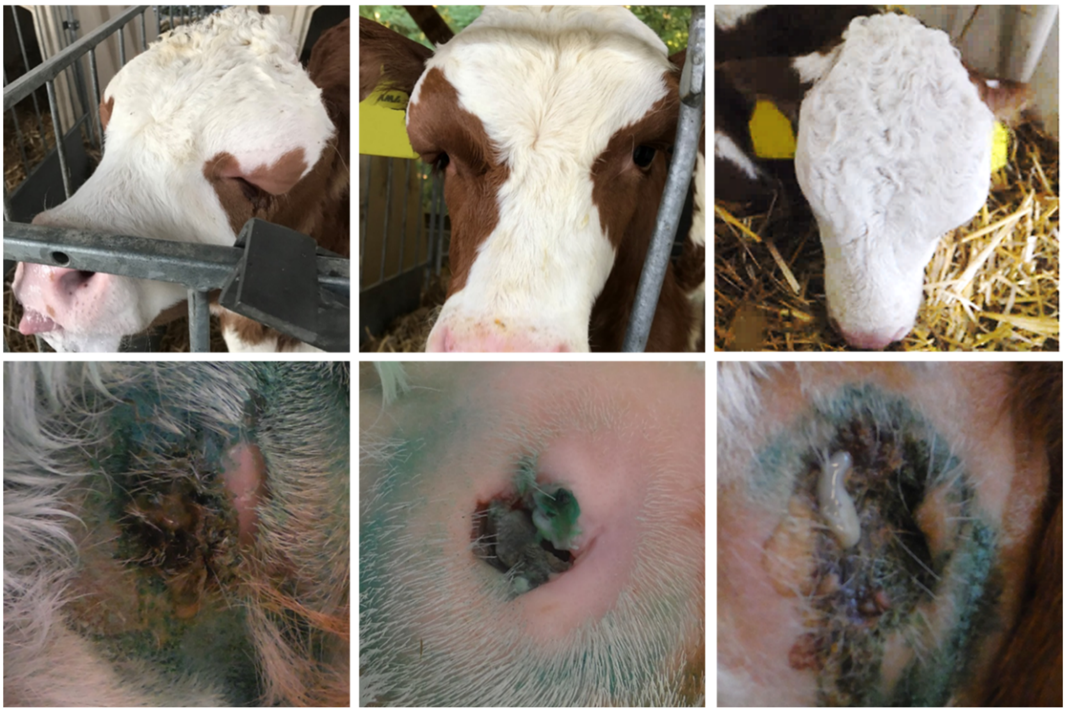

Figure S2. Local reactions after injection of clove oil or isoeugenol. Top: swellings of the upper eyelids on the day after the treatment. The left and centrally picture show severe swellings of both eye lids due to the injection of clove oil, whereas the right one shows a moderate swelling of the left eye lid due to the injection of isoeugenol. Bottom: suppurations on day 21 (left: before cleaning the wound, centrally: after cleaning; clove oil) and day 27 (right, isoeugenol) after the treatment.

Table S1. Tissue alterations assessed during the clinical inspection and palpation of calves with photographs, definitions and scoring. The calves were examined once per week as long as they stayed at the farm. Bold font indicates the different categories in each variable.

Desquamation<br>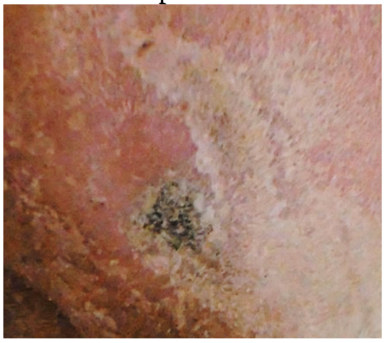<br>Cornified and detached epidermal cells [1]<br>**yes, no**	Discoloration<br>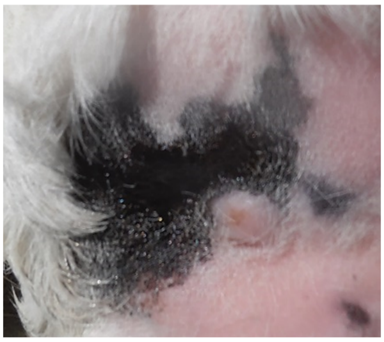<br>Different colour of the skin compared to the normal skin<br>**black** (picture), **red, yellowish, others, no**	Exudate<br>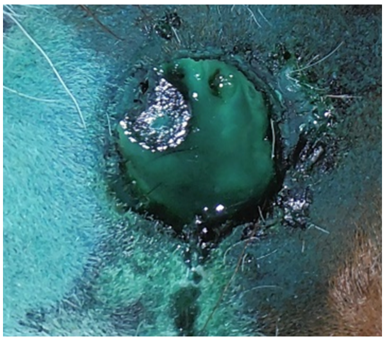<br>Fluid that leaks from the vessels during inflammation and can be more or less cloudy [2] (in the picture it is blue due to the Cyclo spray)<br>**yes, no**
Granulation<br>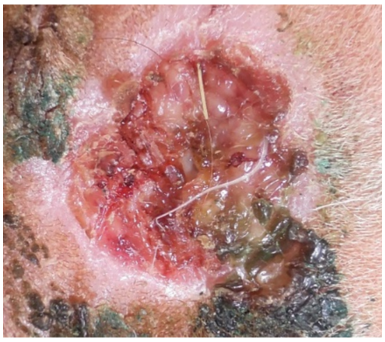<br>Reddish, soft and eventually slightly bleeding warts ("granules") on free surfaces [2]<br>**yes, no**	Incrustation<br>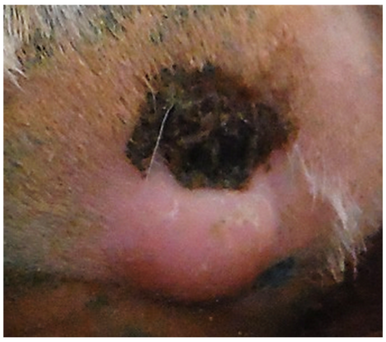<br>Dried exudate, usually with additions of hair, epidermis cells and tissue components [1]<br>**yes, no**	Suppuration<br>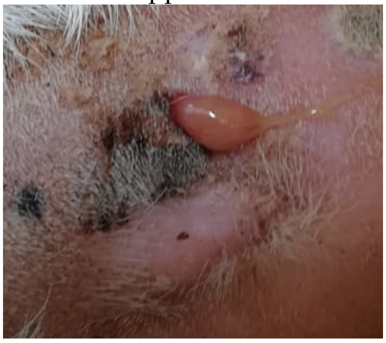<br>Fluid that is white to yellowish in colour and often creamy [1]<br>**yes, no**
Necrotic-like tissue (NLT)<br>Local tissue destruction, likely caused by necrosis<br>**type I, type II, no**	NLT type I<br>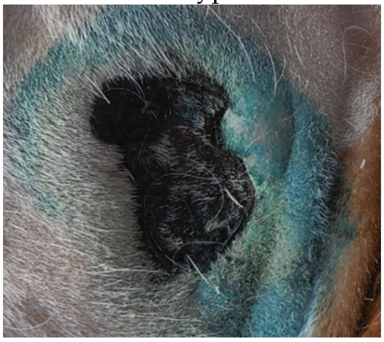<br>Local tissue destruction including sinking of the tissue and a black discoloration (similar to a gangrene), sometimes including a suppuration	NLT type II<br>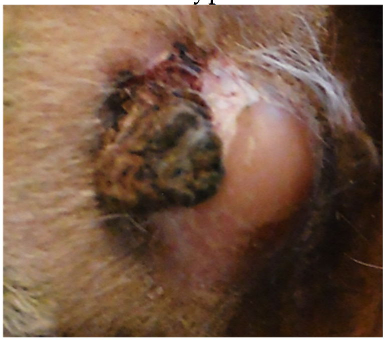<br>Local tissue destruction with tissue growing outside visually similar to a crust, although of a different consistency (similar to firm tissue)

Tissue retraction	Swelling of eye lid	Swelling around horn bud
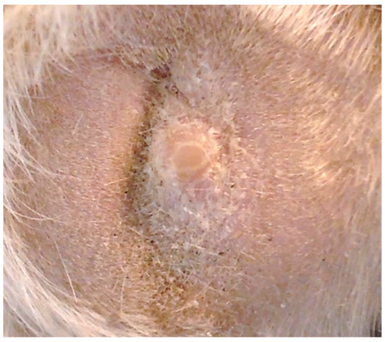	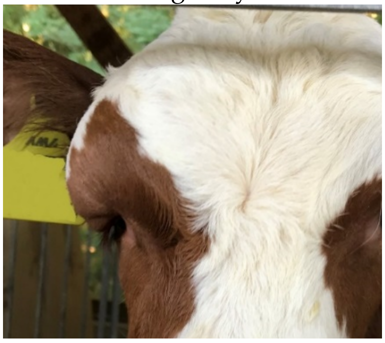	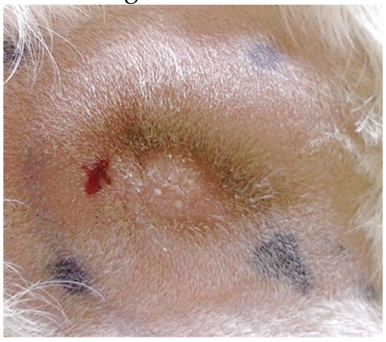
Sinking of the tissue (most likely due to tissue destruction) **yes, no**	Enlargement of the eye lid, sometimes including the area above/around the eye, sometimes followed by lichenification **mild, moderate, severe, lichenification, no**	Enlargement of the tissue around the horn bud, consistency of tissue firmer as unaffected tissue **mild, moderate, severe, no**

[1] modified after Baumgartner [1]; [2] modified after Anonymous [2].

Table S2. Results of the linear mixed model for the mechanical nociceptive threshold measured with von Frey filaments (vFF) and a pressure algometer (PA) in calves of the four different treatments (injection of saline, clove oil or isoeugenol or the disbudding with a hot-iron) at various time points within the first 3 weeks after the treatment.

Factors	F value	Degrees of Freedom	Level of Significance
		vFF	
Treatment	6.626	3/32	0.001
Time point	12.259	8/45	<0.001
Treatment*time point	2.966	23/49	0.001
Age[1]	0.236	1/32	0.631
Treatment*age[1]	5.005	3/32	0.006
		PA	
Treatment	2.067	3/26	0.129
Time point	4.134	8/50	0.001
Treatment*time point	2.008	23/50	0.020
Sex	0.880	1/27	0.356
Time point*sex	2.374	8/50	0.030
Age[1]	0.005	1/27	0.946
Treatment*age[1]	3.031	3/26	0.047
Time point*age[1]	1.857	8/52	0.087

[1] corresponds to the calf's age at treatment (1 to 5 days old).

Table S4. Horn height measured with a hollow cylinder and a slide gauge in calves of the three injection treatments—injection of saline (CON), clove oil (CLOV) or isoeugenol (ISO). Data is shown over all time points (treatment) or for each time point, i.e. before treatment or the indicated days after treatment depicted by the interaction treatment*time point. The fourth treatment group (disbudding with a hot iron) is not included here, as no horn growth was observed. The estimated means and the standard error are presented in mm. The maximum sample size was 20 horns for all treatments (2 horns per animal) per time point. Due to tissue alterations and calves leaving the farm the actual sample size was as follows: CON: 14/20/20/20/18/16/10; CLOV: 16/17/13/18/19/17/12; ISO: 16/20/20/19/20/16/10.

Factors	CON	CLOV	ISO
Treatment	1.00 ± 0.054	0.67 ± 0.052	0.69 ± 0.052
Treatment*time point			
before	0.61 ± 0.055	0.64 ± 0.052	0.61 ± 0.052
14 d	0.73 ± 0.065	0.49 ± 0.066	0.49 ± 0.064
28 d	0.90 ± 0.056	0.58 ± 0.057	0.62 ± 0.054
42 d	1.05 ± 0.059	0.64 ± 0.058	0.71 ± 0.057
56 d	1.14 ± 0.062	0.73 ± 0.060	0.75 ± 0.059
70 d	1.23 ± 0.063	0.76 ± 0.061	0.77 ± 0.062
84 d	1.32 ± 0.086	0.87 ± 0.080	0.85 ± 0.085

References

1. Baumgartner, W. *Klinische Propädeutik der Haus—und Heimtiere*; 8th ed.; Enke Verlag: Stuttgart, Germany, 2014, 64-71.
2. Anonymous. Roche medical lexicon; 5th ed.; Elsevier GmbH, Urban & Fisher Verlag: Munich-Jena , Germany, 2003. Available online: https://www.roche.de/lexikon/index.htm?userInput=Suche%2520im%2520Roche%2520Lexikon&loc=www.roche.de (accessed 20 July 2020).
